# A Delphi Study on the Changes in Work, Organizational Culture, and Health Issues of Nurses at Tertiary Hospitals in South Korea during the COVID-19 Pandemic

**DOI:** 10.1155/2024/9421360

**Published:** 2024-09-04

**Authors:** MiRa Yun, WonJong Kim, Boas Yu, Eun-Hi Choi

**Affiliations:** ^1^ Department of Nursing Sangmyung University, 31, Sangmyeongdaegil, Dongnam-gu, Cheonan-si, Republic of Korea; ^2^ Department of Nursing Gimcheon University, Gimcheon-si 39528, Republic of Korea; ^3^ Henry P. Becton School of Nursing and Allied Health Fairleigh Dickinson University, Madison, New Jersey 07940, USA; ^4^ College of Nursing Eulji University, Uijeongbu-si 11759, Republic of Korea

## Abstract

Nurses in South Korea experience high work intensity and poor working environments, which worsened during the COVID-19 pandemic. This study aimed to evaluate the work changes and grievances of nurses who provided direct care for patients at tertiary hospitals during the pandemic. The nurses' perceptions of their organizational culture and its impact on nurses' health were also explored. A three-round Delphi study was conducted with 36 expert group participants from six South Korean tertiary hospitals. Overall, 36, 35, and 33 participants responded in the first, second, and third rounds, respectively. Nursing work was divided into three categories: “work related to COVID-19-positive and close contacts,” “work related to COVID-19 negative patients,” and “work related to common nursing tasks.” Organizational culture had the highest average for “increased compliance,” followed by “increased conflict,” “decreased collegiality,” and “growing sense of community.” The identified health problems of nurses during the pandemic were the highest for physical health, followed by mental and social health. These results showed that the workload and work intensity of hospital nurses increased significantly, and their physical, mental, and social health deteriorated during the pandemic. To overcome the crisis, the nursing organizational culture had a strong inclination to comply with the COVID-19-related guidelines with an increased sense of community. As conflicts between employees and departments grew, these were able to be overcome through trust and communication between departments, in which the nursing leadership played an important role. To protect the health and lives of people, it is important to secure skilled nurses in preparation for future disasters. In addition, support is needed to protect the safety and health of nurses and to cultivate effective nursing leadership.

## 1. Introduction

The World Health Organization declared the coronavirus disease 2019 (COVID-19) outbreak a global pandemic in March 2020 (WHO, 2020) [1]. The pandemic had significantly impacted politics, economy, society, culture, and healthcare worldwide, with diverse quarantine policies reflecting the community characteristics of each country [2, 3], as the COVID-19 continued in a rapid mass-spread pattern [4]. Nurses were at the forefront of this crisis, facing numerous challenges (i.e., insufficient resources, lack of personal protective equipment, mandated long working hours, and lack of manpower) [5–7].

South Korea's COVID-19 pandemic management received much attention globally as a relatively successful quarantine effort compared to that of other overseas countries, where the number of confirmed cases and deaths increased rapidly [8, 9]. South Korean health authorities quickly established specialized COVID-19 hospitals and recruited medical personnel to treat patients with COVID-19. However, as the pandemic continued and the specialized hospitals ran out of beds, the role of tertiary hospitals expanded to include tracking, isolating, and treating the COVID-19 patients [10].

Therefore, during this period, the work of tertiary hospital nurses became challenging by the direct care of patients with COVID-19, and complex and burdensome indirect care tasks (such as monitoring to prevent patients and their caregivers becoming infected, environmental management according to the quarantine guidelines, and adaptation/implementation of frequently changing COVID-19 guidelines), in addition to the general tasks previously performed. In most South Korean medical institutions, nurses experienced various unexpectedly complex and difficult situations in preventing the spread of infectious diseases [11, 12]. South Korean nurses were called “heroes” during the pandemic and recognized as the main players of the “K-quarantine.” However, meaningful and realistic support was lacking regarding the nurses' grievances and the nurses could not adequately solve their grievances [13, 14].

Before the pandemic, South Korean hospitals were notorious for poor working conditions due to the high job demands and lack of resources for nurses [15]. The COVID-19 pandemic has placed huge physical and psychological burdens on nurses who already have enough on their plates, leading to various health problems. The deterioration of nurses' working environment has raised concerns about worsening occupational health and safety, physical and mental exhaustion, and other complaints such as physical symptoms (i.e., musculoskeletal strains and pain) during the pandemic [16]. In addition, nurses' psychological distress was extremely increased as they were dealing with fears of self-infection and concerns about possible viral transmission to their own families, as well as the soaring work demands while witnessing patient suffering and death [17, 18].

While coping with these unprecedented challenging situations in medical institutions, organizational culture also changed rapidly [19]. Organizational culture significantly influences the working environment and therefore substantially affects the behavior and well-being of medical professionals. However, it is difficult in uniformly evaluating the responses of medical institutions and their impact on nurses because the medical organization environments and policies varied worldwide, and the responses to COVID-19 also differed [20]. As nurses struggled to cope with an unprecedented pandemic, changes in their work and organizational culture and in-depth understanding of their health concerns are essential for allocating manpower and resources and for preparing systems for future disasters. However, few studies have focused on the work of nurses in Korean healthcare settings.

Therefore, using the viewpoints of nurses who provided direct care during the pandemic, this study was conducted to identify changes and related grievances in their work role and organizational culture and identify their health issues. We hope to provide some beneficial data for establishing efficient strategies, such as efficient allocation of manpower and resources and system overhaul, to help stakeholders prepare for future disaster situations.

## 2. Materials and Methods

### 2.1. Research Design

This survey study derived its results by applying the expert group Delphi technique to understand the COVID-19-related changes in work, organizational culture, and health effects as experienced by nurses who worked at tertiary hospitals in South Korea. The Delphi technique is based on the principle of quantitative objectivity that “opinions of two people are more accurate than that of one person” and the principle of democratic decision-making that “many judgments are more accurate than a few,” when accurate information is lacking on the problem to be estimated [10]. The Delphi technique is a method used to investigate the opinions or judgments of experts in the fields of decision-making, prioritization, and prediction [21].

### 2.2. Participants

The number of experts can vary from study to study [22], but the majority of Delphi studies have used between 15 and 20 respondents [23]. However, this study aimed to recruit a total of 40 participants, considering the similarities and differences related to the characteristics of the region and hospital size. The participants were nurses from six tertiary hospitals: three in Seoul, the largest city in South Korea, and three in Daejeon City, a medium-sized metropolitan area. The selected hospitals were representative tertiary hospitals in each region, each with more than 20 clinical departments.

The inclusion criteria for participants were head nurses, senior nurses, or general nurses with preferably at least five years of experience or those with a master's degree or higher, even if their experience was slightly shorter. Nurses working in COVID-19 dedicated wards, intensive care units, operating rooms, outpatient departments, or those in managerial positions not involved in direct patient care were excluded.

Participants were recruited using the snowball sampling technique, and 36 participants agreed to participate after being individually contacted by the researchers. Among the 36 participants, one had less than five years of experience but was included due to holding a master's degree and serving as a senior nurse in the ward.

The response period for each round was set to two weeks. After sending out the questionnaire, responses were awaited for one week, followed by a reminder message to nonrespondents. Another reminder was sent two days before the deadline to encourage responses. Despite these efforts, there were four nonrespondents in the first round, one in the second, and two in the third, resulting in final responses from 36 participants in the first round, 35 in the second, and 33 in the third. All the participants were women with an average age of 35.3 ± 7.9 years. The average nurse experience was 12.7 ± 8.1 years and included staff nurses (44.4%), charge nurses (41.7%), and unit managers (13.9%) in the medical (38.9%) and surgical (61.1%) wards. The levels of education included bachelor's degrees (38.9%), master's (or currently attending) degrees (52.8%), and doctorate (or currently attending) degrees (8.3%) (Table 1).

### 2.3. Data Collection

The research procedures to identify the COVID-19-related changes in nurses' work, organizational culture, and nurses' health in tertiary hospitals were largely divided into draft development and preliminary survey, first Delphi survey and focus group interviews, structured questionnaire development, and main survey.

### 2.4. Draft Development

The researchers reviewed various literature and available data on the work and organizational culture of medical hospital personnel using keywords, such as “inpatient ward,” “work role,” “nurse,” “organizational culture,” and “COVID-19.” The questionnaire was largely composed of three categories: changes in nurses' work, changes in organizational culture, and COVID-19-related effects on health.

Under the category of “changes in nurses' work,” the following open question was asked, “What nursing tasks have changed (in the amount or intensity of work) due to COVID-19?” For the category of “changes in organizational culture,” the following open question was asked, “What has improved and worsened?” The participants were asked to describe perceived changes in detail. Then, they were asked about their health while coping with the COVID-19 at work.

### 2.5. Preliminary Investigation

The preliminary survey was conducted on June 25, 2022, to develop questions, and five participants were selected to conduct the survey. The answers were reviewed to determine if the questions seemed ambiguous, if there were any duplicate questions, or if any items seemed inconsistent with the study purpose. Based on the results of the preliminary survey, the researchers revised the questions.

For example, the questions under “changes in nurse's work” were divided into nursing work prior to and during the COVID-19. The questions about “changes in organizational culture” were restructured as the atmosphere within the organization, communication methods, and efforts to resolve conflicts. Finally, the health questions were reorganized by addressing emotional, physical, and social health aspects.

### 2.6. Delphi Investigation

The first survey was sent via SNS or text message to 40 consenting participants who provided answers within two weeks. In efforts to better understand the results of the first survey, the researchers conducted a focus group interview with three participants who responded to the first round.

After the focus group, the questions were then refined to form a structured questionnaire.

For example, “changes in nursing work” was largely divided into *work related to COVID-19-negative patients*, *work related to COVID-19-positive patients (and other close contact cases)*, and *work related to common nursing tasks*. As the South Korean government's COVID-19 quarantine policy changed periodically, the time period pertaining to nursing work was limited from 2021 to the time of data collection (the third Delphi survey was completed on September 30, 2022).

To assess changes in organizational culture, the questions were divided into nursing floor (ward) atmosphere, communication, and conflict resolution, and the COVID-19-related health effect was divided into physical health, mental health, and social health.

An objective tool was developed to evaluate the frequency and degree of demand for each configured category on a five-point scale. For example, specific items under “changes in nursing work” were rated on a five-point Likert scale (1 = almost none, 2 = occasional implementation, 3 = normal, 4 = do it often, 5 = do it very often). The frequency of work performance and task difficulty were evaluated (1 = very easy, 2 = easy, 3 = normal, 4 = difficult, and 5 = very difficult). The changes in organizational culture and COVID-19-related health effect were also rated (1 = very disagreeable, 2 = disagreeable, 3 = normal, 4 = agree, and 5 = agree very much).

### 2.7. Ethical Considerations

This study was conducted after receiving approval from the Bioethics Committee of E University (IRB no. EU22-29). The participants were informed of the study purpose and procedures and provided written informed consent. In addition, the researchers explained that the survey data would be used only for research purposes and that anonymity and confidentiality would be guaranteed. Participants were informed of their right to refuse and withdraw from the study at any time without any consequences. For each Delphi survey conducted, sufficient time was provided to respond to the survey. A small gift was provided to the participants.

### 2.8. Data Analysis

In principle, the Delphi investigation process is conducted until experts reach an agreement [24] and up to third rounds were performed in this study. The mean and variation coefficients were determined for performance frequency, task difficulty, and degree of consent for each question. The coefficient of variation (CV) is used to evaluate the stability of the question, in which the experts' consensus is high for values <0.5, while values of 0.5–0.8 and ≥0.8 mean that further Delphi investigations are needed because of low agreement [25]. In this step, investigation after the second step was meaningless if an expert consensus is reached [23]. The present study investigated the opinions of the expert panel of nurses using structured questions until there was a consensus reached. The survey was conducted by delivering the questionnaire online to the experts, who responded within 14 days. Open questions were used in the preliminary survey to indicate specific opinions or items to include in the second and third rounds of the survey. Data were analyzed using IBM SPSS Statistics for Windows, version 26.0.

## 3. Results

### 3.1. Frequency and Difficulty of Nursing Work Related to COVID-19

The nursing work related to COVID-19 was largely classified into three categories: *work related to COVID-19-positive patients (and other close contact cases), work related to COVID-19-negative patients,* and *work related to common nursing tasks* (Table 2).

### 3.2. Work Related to Patients Positive for COVID-19 and Close Contact Patients

The nursing work related to COVID-19-positive patients (and other close contact cases) pertains to performing specific tasks for those patients with confirmed COVID-19 or classified as close contact cases in the ward. In this category, “direct nursing work” rather than “indirect nursing work” increased overall. “Environmental and commodity management,” such as cleaning and disinfecting rooms, also increased significantly.

The most frequently performed task related to “direct nursing work” was following the quarantine guidelines including retrieving the COVID-19 results for the patients and guardians (*M* = 4.79, CV = 0.10), which was identified as the second highest degree of difficulty (*M* = 4.52, CV = 0.13). The most difficult task was wearing protective gear when dealing with isolated patients or responding to call bells (*M* = 4.67, CV = 0.12), indicating that the frequent donning and removing of level D protective gear during each patient encounter was very demanding. The added-on tasks of collecting blood and other specimens directly (*M* = 4.00, CV = 0.25; *M* = 4.36, CV = 0.21) and serving/assisting with meals (*M* = 4.15, *C* = 0.18; *M* = 4.03, CV = 0.22) had to be performed by nurses themselves.

The most demanding task in indirect nursing was responding to complaints from patients and guardians following their COVID-19 confirmation after hospitalization (*M* = 4.61, CV = 0.12). As they were often asymptomatic and became positive while in the hospital, addressing complaints, providing explanation in the event of the confirmation or close contact case designation (*M* = 4.55, CV = 0.11), and confirming the change of guidelines for the patients with close contact designation (*M* = 4.21, CV = 0.19) were often difficult and time-consuming.

Environmental and product management work also increased significantly and was noted as difficult. The frequency of performance (*M* = 4.58, CV = 0.12) and difficulty (*M* = 4.52, CV = 0.13) were high for all related procedures, such as securing transport routes for inspection and isolation room. Managing the hospital room was also to be performed frequently and was a demanding task, including transporting confirmed patients and cleaning the hospital room (*M* = 4.36, CV = 0.18; *M* = 4.42, CV = 0.14), room ventilation, disinfection, linen, and curtain management (*M* = 4.48, CV = 0.17; *M* = 4.15, CV = 0.19). In addition, product/item procurement and room management for patients and guardians occurred frequently and were time-consuming, This included the direct procurement of drinking water and ice packs/hot packs for quarantined patients (*M* = 4.15, CV = 0.25; *M* = 4.24, CV = 0.17), room organization and arrangement of personal items as per patients' request (*M* = 4.33, CV = 0.21; *M* = 4.21, CV = 0.18), and management of resident guardian/couriers and food (*M* = 4.33, CV = 0.19; *M* = 4.00, CV = 0.21). In addition, the task of medical and general waste management was also performed frequently (*M* = 4.52, CV = 0.16) and was physically intensive work (*M* = 4.21, CV = 0.17) as some hospitals required nurses to handle medical and general waste due to access restrictions of janitorial staff.

### 3.3. Work Related to COVID-19-Negative Patients

Work related to COVID-19-negative patients refers to work that was increased or added on due to the pandemic. In this category, “indirect nursing work” increased more than “direct nursing work,” and the degree of demand was much higher. Among indirect tasks, the highest degree of demand was an increase in patient status inquiries due to the prohibition of visitors (*M* = 4.64, CV = 0.12) and the frequency of this task performance was also high (*M* = 4.73, CV = 0.10).

There were increased frequencies of reporting on patients and resident guardians' COVID-19 symptom status (*M* = 4.85, CV = 0.09), and the degree of demand was also high (*M* = 4.33, CV = 0.17). This increase in reporting was linked to the increasing number of vital sign measurements for patients and their resident guardians (*M* = 4.21, CV = 0.25; *M* = 3.97, CV = 0.26) because nurses had to monitor not only the patients but also their resident guardians for symptoms and vital sign measurements. In addition, the frequency was high for increasing ward tours for patient safety and fall prevention (*M* = 4.36, CV = 0.15) and the degree of work demand for this task was high (*M* = 4.30, CV = 0.18).

The highest frequencies of direct nursing work were increased COVID-19-related infection and discharge education (*M* = 4.33, CV = 0.21), and the degree of arduousness was high (*M* = 4.27, CV = 0.22). This shows that checking quarantine guidelines, which changed frequently during the chaotic situation of the pandemic; accurately educating patients and guardians; and preventing confusion were the most frequent and arduous tasks.

As the number of patient cases without their resident guardians increased due to the quarantine guidelines, nurses had to perform sanitary management typically performed by the guardians, such as changing diapers and cleaning urine, and both the frequency (*M* = 4.15, CV = 0.25) and the degree of demand (*M* = 4.12, CV = 0.31) were high. In addition, the time per nursing task increased because nurses had to bring only one syringe or a single device at a time for each patient to prevent infection (*M* = 4.27, CV = 0.20), which was too time-consuming and demanding (*M* = 4.09, CV = 0.28).

### 3.4. Changes in Organizational Culture during the COVID-19 Pandemic

#### 3.4.1. Changes in the Nursing Floor (Ward) Atmosphere

Changes in nursing organizational culture were largely divided into changes in the atmosphere of the ward, communication, and conflict resolution measures (Table 3). First, the change in ward atmosphere was classified into four topics, with the highest average observed for *increased rule compliance* (*M* = 4.25), followed by *increased conflict* (*M* = 4.15), *reduced collegiality* (*M* = 3.73), and *growing sense of community* (*M* = 3.58).

The item with the highest average score in *increased rule compliance* was “creating an atmosphere that emphasizes compliance” to the COVID-19 guidelines (*M* = 4.64, CV = 0.11), followed by “eating alone” (*M* = 4.52, CV = 0.14) and “inform/share the frequently changing guidelines and foster interest” (*M* = 4.33, CV = 0.17). To comply with the guidelines, nurses ate alone on their days off (not eating with their families) and during the hospital's working hours. In addition, they refrained from talking but were encouraged to inform/share any changes in guidelines with colleagues.

*Increased conflicts* occurred due to differences in opinions between the department, infection control office, and other departments when transporting confirmed patients (*M* = 4.42, CV = 0.14) and experienced “communication breakdown with other employees” due to work overload (*M* = 4.27, CV = 0.17). The next highest scores were observed for *reduced collegiality*, mostly due to the “increase in alternating nursing assignments for the isolation ward to increase manpower” (*M* = 3.91, CV = 0.32), “decreased personal friendships between employees due to reduced socialization such as ward dinners” (*M* = 3.76, CV = 0.26), and “increased individualism” (*M* = 3.52, CV = 0.34). Some responded that their “sense of community” increased as they came together to overcome the chaotic situations, although the average score was the lowest for this item (*M* = 3.58, CV = 0.32).

#### 3.4.2. Communication and Conflict Resolution Measures

During the COVID-19 pandemic, “non-face-to-face communication through SNS, bulletin boards, videos, and phones” increased (*M* = 4.12, CV = 0.19), while “vertical communication through unit managers” increased (*M* = 3.70, CV = 0.25) and most of the education in hospitals showed “transition to cyber-education, non-face-to-face education, reduced verbal communication” (*M* = 3.55, CV = 0.35).

Regarding conflict resolution measures, the score was highest for: “the attitude of a senior or chief nurse to take the initiative” (*M* = 3.70, CV = 0.31) and “efforts to resolve conflicts between employees through communication” (*M* = 3.70, CV = 0.24). Other conflict resolution measures included: “efforts to understand/sympathize with each other and trust department members” (*M* = 3.67, CV = 0.22), “resolving conflicts through conversations between department heads” (*M* = 3.45, CV = 0.28), “engage in more conversations to find a solution” (*M* = 3.27, CV = 0.33), and “meet with the chief nurse to provide an opportunity to talk about grievances” (*M* = 3.21, CV = 0.35). The lowest score was observed for the item, “avoiding conflict and just moving on” (*M* = 2.76, CV = 0.44).

#### 3.4.3. Health Problems Faced by Nurses during the COVID-19 Pandemic

The most frequently reported health problems faced by nurses during the COVID-19 pandemic were physical health problems (*M* = 4.55), followed by mental health (*M* = 4.33) and social health (*M* = 4.19) problems, with an overall average of 4.35 (Table 4).

The highest physical health score was for “increased fatigue due to frequent shiftwork to cover for infected colleagues” (*M* = 4.82, CV = 0.08). If a previously scheduled nurse was unexpectedly confirmed to be positive for COVID-19, other nurses had to step in to complete the work. The scores for “increased fatigue from increased workload” due to strict quarantine guidelines such as multistep infection prevention measures and wearing protective gear (*M* = 4.76, CV = 0.09) and “feeling sick due to COVID-19 infection” (*M* = 4.70, CV = 0.11) also were high. In addition, even though the self-quarantine period was seven days according to the quarantine guidelines with confirmed COVID-19 infections, “infected nurses could only take five days off and were physically exhausted” (*M* = 4.48, CV = 0.19). The scores for “worsened back pain due to frequent patient turning/position change and increased incontinence care” were also high (*M* = 4.36, CV = 0.20).

The highest mental health score was “increased emotional labor in dealing with guardians and other caregivers” (*M* = 4.76, CV = 0.09), followed by “increased stress due to heavy workloads” (*M* = 4.64, CV = 0.14), “increased anxiety due to the risk of transmission to own families” (*M* = 4.58, CV = 0.12), and “increased stress due to conflicts with guardians and difficulty communicating with patients” (*M* = 4.52, CV = 0.14).

For social health, the item with highest score was “improved societal awareness about nurses during the COVID-19 pandemic” (*M* = 4.48, CV = 0.17). Many participants also reported “decreased usual leisure activities due to increased fatigue” (*M* = 4.45, CV = 0.14) and that “frequent absenteeism due to fellow nurses' infections/illnesses and extended working hours disrupted family life” (*M* = 4.36, CV = 0.17). Many participants also said that they felt “disconnected from social relationships due to social distancing and compliance with quarantine measures” (*M* = 4.18, CV = 0.22) and that they were “socially isolated when they were confirmed to have COVID-19” (*M* = 4.09, CV = 0.24). Additionally, many responded that it was “painful to prioritize and focus only on hospital work” (i.e., forsaking their normal daily lives/routines during the pandemic) (*M* = 3.94, CV = 0.28).

## 4. Discussion

This study was conducted to determine the changes in work roles and complaints of nurses providing direct patient care for inpatients in South Korean tertiary hospitals during the COVID-19 pandemic and to explore nurses' perceptions of organizational culture and nurses' health problem during the crisis. The study showed that Korean nurses experienced increased work intensity, performed multiple tasks without additional staff, and demonstrated various physical, psychological, and social symptoms while coping with the COVID-19 situation.

Korea's COVID-19 pandemic management utilized a risk-control strategy to manage the possibility of risk at an acceptable level using effective containment measures [10]. Accordingly, all people in contact with confirmed patients were tracked, classified as close contacts, and required to follow the quarantine guidelines. Therefore, whenever patients with confirmed COVID-19 or close contact were unexpectedly identified in multibed inpatient rooms, all required multistep nursing activities had to be carried out according to the quarantine guidelines, which were burdensome and complicated tasks.

Direct nursing tasks that required donning protective gear repeatedly and continued compliance with the strict quarantine guidelines increased physical fatigue and burden, while the number and frequency of indirect nursing tasks such as patient transport management and environmental management (i.e., cleaning/sterilizing room and bed) also increased rapidly [11, 26]. The emotional labor of nurses intensified as they addressed perpetual flurry of complaints from both patients and guardians [26].

Due to the insufficient staffing of nurses, most patients in South Korean medical institutions, except those in few hospitals with special (integrated nursing care service) wards, voluntarily have resident guardians who provide care (usually family members or hired help). These guardians perform some of the nonskilled nursing work, such as providing incontinence care and washing/dressing the patients [27]. However, during the pandemic, the number of caregivers on duty was limited due to quarantine requirements. Therefore, nurses had to perform more frequent ward rounds to monitor patient conditions, procure supplies for patients and caregivers, and manage the ward.

Tasks such as changing diapers and assisting with meals (which were typically performed by guardians) became additional tasks for nurses, as the allowed number of guardians was limited due to the COVID-19 guidelines. Other added-on indirect nursing work such as purchasing and delivering of goods (water, snacks, etc.) for quarantined patients also increased. Nurses also had added-on work of other personnel, such as phlebotomist, dietary, and janitorial staff [28]. As most hospitals restricted access to other nonnursing personnel to prevent the spread of infection, nurses had to perform nonnursing work, such as collecting blood and other laboratory specimens, delivering/serving meals, and disinfecting/sterilizing supplies.

In addition, when moving patients with COVID-19, the movement of others in hallways, elevators, and other areas had to be controlled. Therefore, they had to follow strict quarantine procedures, including notifying other patients and caregivers, and contacting facility management. Nurses were also required to provide numerous explanations and make announcements. These include explaining the policies, such as having only one resident guardian per patient; checking and explaining the COVID-19 guidelines when going out/leaving the hospital or visiting; monitoring the proper application of guardians' masks and hospital room curtains, management of guardians' outings, and separation from visitors as needed; and knowing/enforcing infection control guidelines and hospital regulations.

This study illustrates the reality of Korean hospitals, where nurses are expected to perform a myriad of patient-related tasks, including the role of doctors, phlebotomist, nursing assistants, and clinical pathologists, as well as the role of room management, transport, and cleaning services, without any specific job role limitations. Nurses in South Korean hospitals have expressed distress regarding the inability to eat, use the bathroom, and engage in whatever task they have to perform [15]. Distress among nurses gained attention during the COVID-19 pandemic.

This is also a problem with the current legal system, where the role of nurses is not clearly defined in the law but only mentioned in a single line in the Medical Service Act revised in 1973, “nursing or medical attendance of the sick or pregnant women” [29]. Although in Korea, the role of nurses over the past 5 years has become more sophisticated and broader in scope with the rapid development of medical technology, the law has not been updated, creating a myriad of problems [30]. The definition of the role of nursing is unclear; therefore, the advanced tasks performed by nurses cannot be verified whether they are legal, but all the tasks in hospitals can easily be transferred to nurses.

In addition, the ratio of patients to nurses in Korean hospitals is high by default. A study reported that the patient per nurse ratio (16.3 patients per nurse) was very high and was 2-3 times higher than that of either the United States or the United Kingdom [31]. Even before COVID-19, it was reported that 63.2% of nurses in South Korean hospitals skipped meals more than once a week due to workload, 48.2% did not have annual leave/vacation, and 81.0% were at high risk for accidental injuries due to insufficient staffing [32]. The COVID-19 pandemic has exacerbated the poor working conditions of these nurses.

Another challenge for nurses was the inability to take time off for holidays or weekends due to the frequent shift changes. For example, it was common for an off-duty nurse to cover for a colleague who could not report to work due to an unexpected COVID-19 case. Despite the quarantine guidelines of self-quarantining for 7 days in the event of COVID-19, nurses often had only five days off before returning to work. The reason was that the hospital was not staffing enough nurses and was running shifts with fewer staff, so no nurses were available to cover the vacant duties. Nurses became exhausted and often experienced symptoms, such as physical pain, fatigue, depression, anxiety, and posttraumatic stress disorder because of long hours of high-intensity labor, lack of rest, and limited manpower [33–35]. If no nurses were available to replace them, the workload increased due to overwork. If a nurse suddenly resigned midshift, the remaining nurses suffered with a heavier workload [11, 17].

These poor working conditions during the pandemic contributed to nurses' health problems, which were not only physical and psychological symptoms due to the stress and strain at work, but also social, such as limited contact with family and friends to protect patients. With less time and opportunities for stress relief and self-care, prolonged negative emotional and psychological states can affect nurses' mental health and quality of nursing [36]. Therefore, systematic coping support for nurses to meet their physical and mental needs is important [37], as well as providing reasonable job accommodations (i.e., flexible shiftwork schedules, appropriate time-off for proper rest and self-care) [7, 38]. However, such support systems are rare in Korea.

As a result of this poor working environment, the resignation rate of Korean nurses is increasing yearly. In 2018, 42.7% of new nurses working in tertiary or general hospitals resigned within a year of hire; this number surged to 52.8% in 2021 (during the COVID-19 outbreak) [39]. Currently, the average year worked for hospital nurses in South Korea is only seven years and eight months, with 52.1% having less than five years of experience [40]. A sufficient number of well-skilled and experienced nurses are important to provide high-quality nursing, especially in the case of future disasters. However, during the COVID-19 response, a major issue in South Korea's disaster response system was identified as the availability of human resources [41].

To address the shortage of nurses, the Korean government has promoted a policy of continuously increasing the number of admissions to nursing schools. Therefore, the number of nursing admissions in Korea has increased to the highest among Organization for Economic Cooperation and Development (OECD) countries, although the number of nurses actually working in clinical practice is lower than the OECD average [42, 43]. In other words, most graduate nurses cannot bear the poor reality with the nature of the work and eventually leave the field. To ameliorate this problem, the Nursing Workforce Human Rights Act, which stipulates the number of patients per nurse, was proposed by civil society organizations [44], and the Nursing Act, which stipulates the role and scope of nurses, passed the National Assembly with difficulty, but was not funded and abandoned due to the medical associations, various interest groups, and political reasons [45]. The COVID-19 pandemic has shown that policy and institutional changes to maintain a stable workforce of qualified nurses are very urgent and can no longer be postponed.

Meanwhile, the biggest change in the organizational culture of nurses was “compliance with COVID-19 guidelines,” which has emerged as top priority. Such strict compliance was an effort by nurses to control dangerous situations as much as possible. Previous studies showed that nurses implemented infection control measures more strictly than others to prevent the spread of infection and to limit contact not only between medical personnel in hospitals but also with own family members [11].

In addition, conflicts between employees and departments increased amid confusion, with a breakdown in communication. Face-to-face meetings disappeared and collegiality between employees decreased as they mainly communicated non-face-to-face. However, the leadership of nurses in charge or the unit managers was mentioned as the driving force in resolving conflicts, and difficult situations were overcome through dialogue and trust between departments [11, 17].

Studies demonstrate that effective nursing leadership is critical for disaster response. In a study of nursing leadership in South Korea during the COVID-19 pandemic, Oh et al. found that nursing leaders played an important role in providing clear communication, facilitating team collaboration, and allowing nurses to access appropriate protective gear and resources [46]. The authors argued that nursing leaders should possess the skills necessary to effectively manage and support clinical teams.

In summary, hospital nurses in South Korea experienced physical, psychological, and social health problems during the pandemic as they endured increased work intensity, insufficient leave, inadequate support and resources, and a lack of legislation on their role. However, through their dedication, leadership, and commitment to following guidelines, nurses have overcome the challenges.

The unprecedented COVID-19 pandemic was very challenging and difficult for medical personnel worldwide. During the pandemic, nurses worldwide faced numerous challenges, such as fear of contagion, burnout from excessive work, physical fatigue, moral pain, and high level of stress [7, 47–49]. However, in past disasters, altruistic nurses overcame crises with a sense of vocation [50, 51]. Nurses strengthened their sense of solidarity and formed cooperative relationships in dealing with the COVID-19 [8, 9, 40]. The positive organizational culture and atmosphere played a pivotal role in overcoming adversities or difficult challenging situations by enhancing individual nurses' responsibilities for human-centered care [11, 13, 52].

Based on the study findings, we suggest the following.

First, healthcare laws and regulations should be revised and overhauled by effectively reducing patient-nurse ratios and legally clarifying nurses' professional roles to reduce excessive workloads and burdens on nurses.

Second, approaches should be explored to improve the organizational culture of nursing and the leadership of nurse managers.

Third, a national support system for nurses' mental and physical health is needed.

### 4.1. Strengths and Limitations

This study collected data from 36 nursing experts from six tertiary hospitals nationwide in South Korea. Owing to the limitations in sampling, these results cannot be generalized. Another limitation is that the data were derived from the perspective of nurses and do not reflect the views of the whole hospital, including other professions. Nevertheless, using the Delphi technique, we obtained a consensus from the recruited experts regarding the changes in work, organizational culture, and health problems faced by nurses working in top tertiary hospitals during the pandemic. It is also meaningful in that it provides a picture of the realities faced by nurses based on their first-hand account of the work crisis.

### 4.2. Implications for Nursing Management

This study has shown that, despite the many challenges Korean nurses faced, their sacrifices were vital to the nation's efforts to overcome the pandemic. It has also highlighted the problems caused by the lack of adequate staffing and a clear legislation on the role of nurses. In conclusion, this study suggests that policies to address the legal and institutional deficiencies related to the nursing workforce are urgently needed to prepare for future disasters and protect public health and lives.

In the future, nursing educators and clinical professionals should actively discuss and conduct research on the appropriate deployment of nursing staff, defining the scope of nurses' roles and duties and improving the working environment for nurses. Concurrently, they should continue to demand legal and institutional improvements from hospital administrators and the state. Research should also focus continually on developing nurses' leadership skills.

## 5. Conclusions

The results of this study showed that the workload and work intensity of South Korean hospital nurses increased significantly, and their physical, mental, and social health deteriorated during the COVID-19 pandemic. The reason for this was the unprecedented crises and related confusion caused by the pandemic itself, as well as the characteristics of South Korean medical institutions. Due to the high patient to nurse ratio, the intensity of nurses' work is too high. This is further compounded by the fact that role of nurses is not legally defined, which placed nurses in disadvantaged position and became worse during the pandemic. However, to overcome the crises, the nursing organizational culture had a strongly supportive atmosphere of the guideline compliance with an increased sense of community. Even though conflicts between employees and departments grew as the pandemic persisted, these were resolved based on trust and communication between departments, with effective nursing leadership playing an important role.

## Figures and Tables

**Table 1:** General characteristics of the expert panel (*N* = 36).

Variables	Categories	*n* (%) or *M* ± SD
Sex	Female	36 (100.0)

Age (years)	<30	11 (30.6)
	30–39	16 (44.4)
	40–49	6 (16.7)
	50≤	3 (8.3)
	*M* ± SD	35.3 ± 7.9

Experience (years)	<5	1 (2.7)
	5–9	15 (41.7)
	10–19	15 (41.7)
	20≤	5 (13.9)
	*M* ± SD	12.68 ± 8.1

Position	Staff nurse	16 (44.4)
	Charge nurse	15 (41.7)
	Unit manager	5 (13.9)

Department	Medical ward	14 (38.9)
	Surgical ward	22 (61.1)

Education	Bachelor's degree	14 (38.9)
	Master's degree	19 (52.8)
	Ph.D. degree	3 (8.3)

**Table 2:** Frequency and difficulty in performing nursing work related to coronavirus disease 2019 (COVID-19).

Category		Item	Frequency		Difficulty	
			Mean	CV	Mean	CV
Work related to COVID-19-positive and close contacts	Direct nursing work	(1) Use protective gear to treat quarantined patients or respond to call bells	4.67	0.12	4.58	0.12
		(2) Multistep nursing activities required according to isolation guidelines until COVID-19 results of patients and guardians are released	4.79	0.10	4.52	0.13
		(3) Direct blood specimen collection by nurses due to access restrictions	4.00	0.25	4.15	0.18
		(4) Serving meals/assisting with meals due to access restrictions	4.36	0.21	4.03	0.22
		(5) Check whether periodic PCR tests are performed and observe symptoms	4.70	0.10	4.33	0.15
	Indirect nursing work	(1) Responding to complaints from patients and guardians	4.61	0.11	4.61	0.12
		(2) Explaining the situation in the event of confirmation or close contact	4.27	0.23	4.55	0.11
		(3) Confirmation of changed guidelines during the examination of patients with close contacts	4.36	0.18	4.21	0.19
	Environmental and commodity management	(1) All procedures, such as securing a transport route for inspection and isolation room	4.58	0.12	4.52	0.13
		(2) Clean the hospital room after quarantine of confirmed patients	4.36	0.18	4.42	0.14
		(3) Direct procurement of drinking water, ice packs, and hot packs for quarantined patients	4.15	0.25	4.24	0.17
		(4) Management of medical and general waste	4.52	0.16	4.21	0.17
		(5) Organize or manage items that are not managed by a single patient	4.33	0.21	4.21	0.18
		(6) Securing beds to move to the hospital room after the quarantine period ends	3.97	0.29	4.15	0.18
		(7) Ventilation, disinfection, linen, and curtain management in the hospital room	4.48	0.17	4.15	0.19
		(8) Assisting guardians (i.e., food and other related management)	4.33	0.19	4.00	0.21

Work related to COVID-19-negative patients	Direct nursing work	(1) Increased COVID-19 inpatient infections and related discharge education	4.33	0.21	4.27	0.22
		(2) Increased hygiene management such as changing diapers and cleaning up urine/feces	4.15	0.25	4.12	0.31
		(3) Bringing syringes one at a time to each patient to prevent infection	4.27	0.20	4.09	0.19
		(4) Increased number of patients without guardians, increasing meal assistance	3.97	0.31	4.00	0.28
		(5) Increased frequency of checking vital sign measurements for patients/guardians	4.21	0.25	3.97	0.26
	Indirect nursing work	(1) Increased calls for patient status due to prohibition of guardian visits	4.73	0.10	4.64	0.12
		(2) Increasing reporting of patients and guardians' COVID-19 symptoms	4.85	0.09	4.33	0.17
		(3) Increased ward tours for patient safety and fall management	4.36	0.15	4.30	0.18
		(4) Increased use of monitors and device rental due to increased patient severity	4.30	0.20	4.06	0.26
		(5) Regular PCR testing and confirmation of results to patients and caregivers, and confirmation of vaccination	4.79	0.11	4.06	0.25
		(6) Increased checklist for COVID-19 testing and infection in surgical patients	4.15	0.31	3.73	0.38
	Environmental and commodity management	(1) Item delivery to patients due to discontinuation of guardian visits and increased purchases	4.03	0.31	3.91	0.27

Work related to common nursing tasks		(1) Management of outings (outings of guardians or caregivers, and separation of movement from other outpatients)	4.39	0.21	4.24	0.21
		(2) Familiarization with changed infection control or in-hospital guidelines	4.70	0.10	4.21	0.15
		(3) Explaining the hospital policy (i.e., one guardian per patient, following the guidelines when visiting or going out)	4.91	0.06	4.12	0.19
		(4) Monitoring the application of resident guardians' masks and curtains, and announcing other relevant restrictions	4.76	0.16	4.12	0.25
		(5) Fever check and other related management with visitors	4.58	0.15	3.88	0.30
		(6) Identifying the number of caregivers or guardians	4.70	0.11	3.85	0.27

**Table 3:** Changes in nursing organizational culture during the coronavirus disease 2019 (COVID-19) pandemic.

Categories		Mean ± SD	Items	Mean	CV
Changes in the atmosphere	Increased rule compliance	4.25 ± 0.44	(1) Creating an atmosphere that emphasizes compliance	4.64	0.11
			(2) Eating alone (while working and at home)	4.52	0.14
			(3) Inform/share the guidelines and foster interest	4.33	0.17
			(4) Only work-related conversations allowed during work	3.52	0.30
	Increased conflict	4.15 ± 0.22	(1) Increased conflicts between the main department, infection control room, and other departments when transporting confirmed patients	4.42	0.14
			(2) Overwork leads to communication breakdown with other employees	4.27	0.17
			(3) Increased sensitivity/reactivity to each other	4.06	0.19
			(4) Work style conflicts as multiple department personnel are mixed	3.85	0.28
	Decreased collegiality	3.73 ± 0.16	(1) Increase in alternating nursing assignments for the isolation ward to increase manpower	3.91	0.32
			(2) Decreased personal friendships between employees due to reduced socialization, such as ward dinners	3.76	0.26
			(3) Increased individualism	3.52	0.34
	Growing sense of community	3.58 ± 0.00	(1) Increasing sense of community to overcome the crisis together	3.58	0.32

Communication and conflict resolution measures	Communication	3.64 ± 0.34	(1) Non-face-to-face communication through SNS, bulletin boards, videos, phone calls, etc.	4.12	0.19
			(2) Vertical communication through the unit manager	3.70	0.25
			(3) Transition to cyber-education, non-face-to-face education, reduced verbal communication	3.55	0.35
			(4) Communication with caregivers and guardians by broadcast	3.18	0.39
	Conflict resolution measures	3.39 ± 0.32	(1) The attitude of a senior or chief nurse to take the initiative	3.70	0.31
			(2) Efforts to resolve conflicts between employees through communication	3.70	0.24
			(3) Efforts to understand/empathize with each other and trust department members	3.67	0.22
			(4) Resolving conflicts through conversations between department heads	3.45	0.28
			(5) Engage in more conversations to find a solution	3.27	0.33
			(6) Meet with the chief nurse to provide an opportunity to talk about grievances	3.21	0.35
			(7) Avoiding conflict and just moving on	2.76	0.44

**Table 4:** Health problems faced by nurses during the coronavirus disease 2019 (COVID-19) pandemic.

Categories	Mean ± SD	Items	Mean	CV
Physical	4.55 ± 0.21	(1) Increased fatigue due to frequent shiftwork to cover for infected colleagues	4.82	0.08
		(2) Increased fatigue from increased workload due to strict quarantine guidelines (multistep protective measures, wearing protective gear, etc.)	4.76	0.09
		(3) Feeling sick due to COVID-19 infection	4.70	0.11
		(4) When infected, nurses could only take five days off and were physically exhausted	4.48	0.19
		(5) Increased musculoskeletal and other physical symptoms due to increased intensity of work and frequently wearing uncomfortable protective gear	4.42	0.16
		(6) Worsened back pain due to frequent patient turning/position change and increased incontinence care	4.36	0.20
		(7) Muscle weakness and decreased physical strength	4.27	0.21

Psychological	4.33 ± 0.39	(1) Increased emotional labor in dealing with guardians and other caregivers	4.76	0.09
		(2) Increased stress due to heavy workloads	4.64	0.14
		(3) Increased anxiety due to the risk of transmission to own families	4.58	0.12
		(4) Increased stress due to conflicts with guardians and difficulty communicating with patients	4.52	0.14
		(5) Difficulty in coping with stress	4.39	0.16
		(6) Increased stress due to insufficient care time per patient due to heavy workloads	4.18	0.20
		(7) Increased loneliness and depression	3.97	0.20
		(8) Low self-esteem (“I studied for 4 years and got a diaper license.”)	3.61	0.30

Social	4.19 ± 0.27	(1) Improved societal awareness about nurses during the COVID-19 pandemic	4.48	0.17
		(2) Decreased usual leisure activities due to increased fatigue	4.45	0.14
		(3) Frequent absenteeism and extended working hours interfered with family life	4.36	0.17
		(4) Disconnected from social relationships to keep social distance and to comply with quarantine measures	4.18	0.22
		(5) Socially isolated when they were confirmed to have COVID-19	4.09	0.24
		(6) Painful to prioritize and focus only on hospital work	3.94	0.28
		(7) Nurses caring for COVID-19 patients were avoided by others	3.79	0.26

## Data Availability

Data used to support the findings of this study are available upon request by e-mail to the corresponding author.

## References

[1] World Health Organization (2020). WHO Director, General’s opening remarks at the media briefing on COVID-19, 11 March 2020. https://www.who.int/director-general/speeches/detail/who-director-general-s-opening-remarks-at-the-media-briefing-on-covid-19---11-march-2020.

[2] Öner H., Arslantaş H., Koruklu N., Sari E., Aslan R. (2022). COVID-19 patients’ life events, emotional health and post-illness awareness: a qualitative study. *Journal of Community Health Nursing*.

[3] Thomas A. S., Osbourne M., Appelhans B. M., Roisman G. I., Booth-LaForce C., Bleil M. E. (2022). Disparities in COVID‐19–related stressful life events in the United States: understanding who is most impacted. *Health and Social Care in the Community*.

[4] Yang Y., Wang H., Chen K., Zhou J., Deng S., Wang Y. (2020). Shelter hospital mode: how do we prevent COVID-19 hospital-acquired infection?. *Infection Control and Hospital Epidemiology*.

[5] Abbas M., Zhu N. J., Mookerjee S. (2021). Hospital-onset COVID-19 infection surveillance systems: a systematic review. *Journal of Hospital Infection*.

[6] Sampaio F., Sequeira C., Teixeira L. (2021). Impact of COVID-19 outbreak on nurses’ mental health: a prospective cohort study. *Environmental Research*.

[7] Chersich M. F., Gray G., Fairlie L. (2020). COVID-19 in Africa: care and protection for frontline healthcare workers. *Globalization and Health*.

[8] Bicker L. (2020). Coronavirus in South Korea: how ‘trace, test and treat’ may Be saving lives. *BBC News*.

[9] Timothy W., Martin T. W., Yoon D. (2020). How South Korea successfully managed coronavirus: the country has blended technology and testing like No other. *Wall Street Journal*.

[10] Lee J. S. (2001). *Delphi Method: Kyoyookgwahaksa. Gyeonggi: Paju*.

[11] Oh H., Lee N. K. (2021). A phenomenological study of the lived experience of nurses caring for patients with COVID-19 in Korea. *Journal of Korean Academy of Nursing*.

[12] Jeon M. K., Son S. Y., Yu J. Y. (2023). Experiences of nurses with COVID-19 infection management in general hospitals and long-term care hospitals. *Crisis and Emergency Management: Theoria and Praxis*.

[13] Lee M. S., Choe M. A. (2021). Nurses fighting on the front lines of COVID-19. *Perspectives in Nursing Science*.

[14] Lim S. M. (2021). COVID-19 field nurses, physical stress is so hard. *Dailymedi*.

[15] Yun M. R., Baek H., Kim I. A., Sung J. M. (2022). Occupational stress in Korean hospital nurses: secondary data analysis of the development of a Korean nurses’ occupational stress scale. *Journal of Korean Academy of Nursing Administration*.

[16] Magnavita N., Tripepi G., Di Prinzio R. R. (2020). Symptoms in health care workers during the COVID-19 epidemic. a cross-sectional survey. *International Journal of Environmental Research and Public Health*.

[17] Chung S. J., Seong M. H., Park J. Y. (2022). Nurses’ experience in COVID-19 patient care. *Journal of Korean Academy of Nursing Administration*.

[18] Shreffler J., Petrey J., Huecker M. (2020). The impact of COVID-19 on healthcare worker wellness: a scoping review. *Western Journal of Emergency Medicine*.

[19] Zhang Y., Dare P. S., Saleem A., Chinedu C. C. (2022). A sensation of COVID-19: how organizational culture is coordinated by human resource management to achieve organizational innovative performance in healthcare institutions. *Frontiers in Psychology*.

[20] Hur J. Y., Kim S. J., Lee J. H. (2021). *Developing a national crisis management strategy for forecasting and responding to emerging risks: a focus on the Covid-19 pandemic*.

[21] Lee E. O., Im N. Y., Park H. (2009). *Nursing Research and Statistical Analysis*.

[22] Delbecq A. L., Van de Ven A. H., Gustafson D. H. (1975). *Group Techniques for Program Planning: A Guide to Nominal Group and delphi Processes*.

[23] Ludwig B. (1997). Predicting the future: have you considered using the Delphi methodology?. *Journal of Extension*.

[24] Rowe G., Wright G., Bolger F. (1991). Delphi: a reevaluation of research and theory. *Technological Forecasting and Social Change*.

[25] Dajani J. S., Sincoff M. Z., Talley W. K. (1979). Stability and agreement criteria for the termination of Delphi studies. *Technological Forecasting and Social Change*.

[26] Lim J. S., Choi Y. S., Kim H. S. (2022). The work performance of infection control nurses in general hospitals during the early COVID-19 pandemic. *Journal of Korean Academy of psychiatric and Mental Health Nursing*.

[27] Kim J. H., Kim S. J., Park E. T., Jeong S. Y., Lee E. H. (2017). Policy issues and new direction for comprehensive nursing service in the national health insurance. *Journal of Korean Academy of Nursing Administration*.

[28] Ko J. M. (2022). *Resident Doctor Works ‘Unlimited’ Nurses Suffer from Work Other than Nursing*.

[29] Ministry of Government Legislation Kr (2023). Revised medical service Act.

[30] Min B. Y. (2022). Nursing law should be enacted to promote public safety and health. *Korean Medicine Newspaper*.

[31] Cho S. H., Lee J. Y. (2016). Changes in nursing workforce and nursing services. *Korean Social Trends 2016*.

[32] (2019). Korea health and medical workers’ union, regular survey on work environment of healthcare workers. https://bogun.nodong.org/xe/khmwu_5_4/543931.

[33] Park S. Y. (2022). Related factors to Korean hospital nurses in burnout during the COVID-19 outbreak: a systematic review. *Journal of the Korea Society of Computer and Information*.

[34] Lee Y. J. (2022). The mediating effect of social support on the relationship between social isolation and depression of nurses in the COVID-19 specialized hospitals. *Journal of Industrial Convergence*.

[35] Gong E. J., Kim J. H. (2022). Influence of fatigue and role-overload on depression in nurses caring for COVID-19 patients. *Journal of the Korea Contents Association*.

[36] Matsumoto Y., Fujino J., Shiwaku H. (2021). Factors affecting mental illness and social stress in hospital workers treating COVID-19: paradoxical distress during pandemic era. *Journal of Psychiatric Research*.

[37] Benton D. C., Alexander M., Fotsch R., Livanos N. (2020). Lessons learned and insights gained: a regulatory analysis of the impacts, challenges, and responses to COVID-19. *OJIN: Online Journal of Issues in Nursing*.

[38] Park S. M. (2021). A review of researches relating to mental health supports for healthcare workers during COVID-19 pandemic. *Taegu Science University Defense Security Institute*.

[39] Lee H. H. (2023). The growth rate of nurses in Korea is four times higher than the OECD Average. *ChosunMedia*.

[40] Song S. Y. (2023). The number of nurses leaving the hospital increases every year. *Excessive Causes of Other Operations*.

[41] Hong S. H., Kim S. B., Ahn H. K. (2021). *Compliance with COVID-19 non-pharmaceutical interventions and regulatory governance in Korea*.

[42] Ministry of Health and Welfare Kr (2018). *Measures to Improve the Working Environment and Treatment of Nurses, Sejong*.

[43] Jang W., Han S. J. (2018). Korea’s medical status and problems according to OECD statistics. *HIRA Policy Trends*.

[44] Doctor Y. (2022). *The Nursing Act Is Not Enough We Need to Enact the Nursing Staffing Standards Act*.

[45] Medical observer (2023). Two-year nursing law battle ends with presidential veto?. https://www.monews.co.kr/news/articleView.html?idxno=323063.

[46] Kirwan S., Cunningham P., Donohue G., Keogh B., Creedon J. (2021). Nursing leadership in response to the COVID-19 crisis in an Irish independent mental health service. *British Journal of Mental Health Nursing*.

[47] D’emeh W. M., Yacoub M. I., Shahwan B. S. (2021). Work-related stress and anxiety among frontline nurses during the COVID-19 pandemic: a cross-sectional study. *Journal of Psychosocial Nursing and Mental Health Services*.

[48] Zheng R., Zhou Y., Fu Y. (2021). Prevalence and associated factors of depression and anxiety among nurses during the outbreak of COVID-19 in China: a cross-sectional study. *International Journal of Nursing Studies*.

[49] Da Rosa P., Brown R., Pravecek B. (2021). Factors associated with nurses emotional distress during the COVID-19 pandemic. *Applied Nursing Research*.

[50] Carter M. (2014). Vocation and altruism in nursing: the habits of practice. *Nursing Ethics*.

[51] Hanna D. R. (2016). A vocation to professional nursing: the intelligence behind compassion. *Research and Theory for Nursing Practice*.

[52] Oh I. O., Yoon S. J., Nam K. A. (2021). Working experience of nurses at a COVID-19 dedicated hospital. *Korean Journal of Adult Nursing*.

